# Supplementary figures and images for: Continuous-time random walk model for the diffusive motion of helicases
Source: QRB Discov. 2025 Oct 9;6:e26. doi: 10.1017/qrd.2025.10011 (PMC12722086; doi:10.1017/qrd.2025.10011)

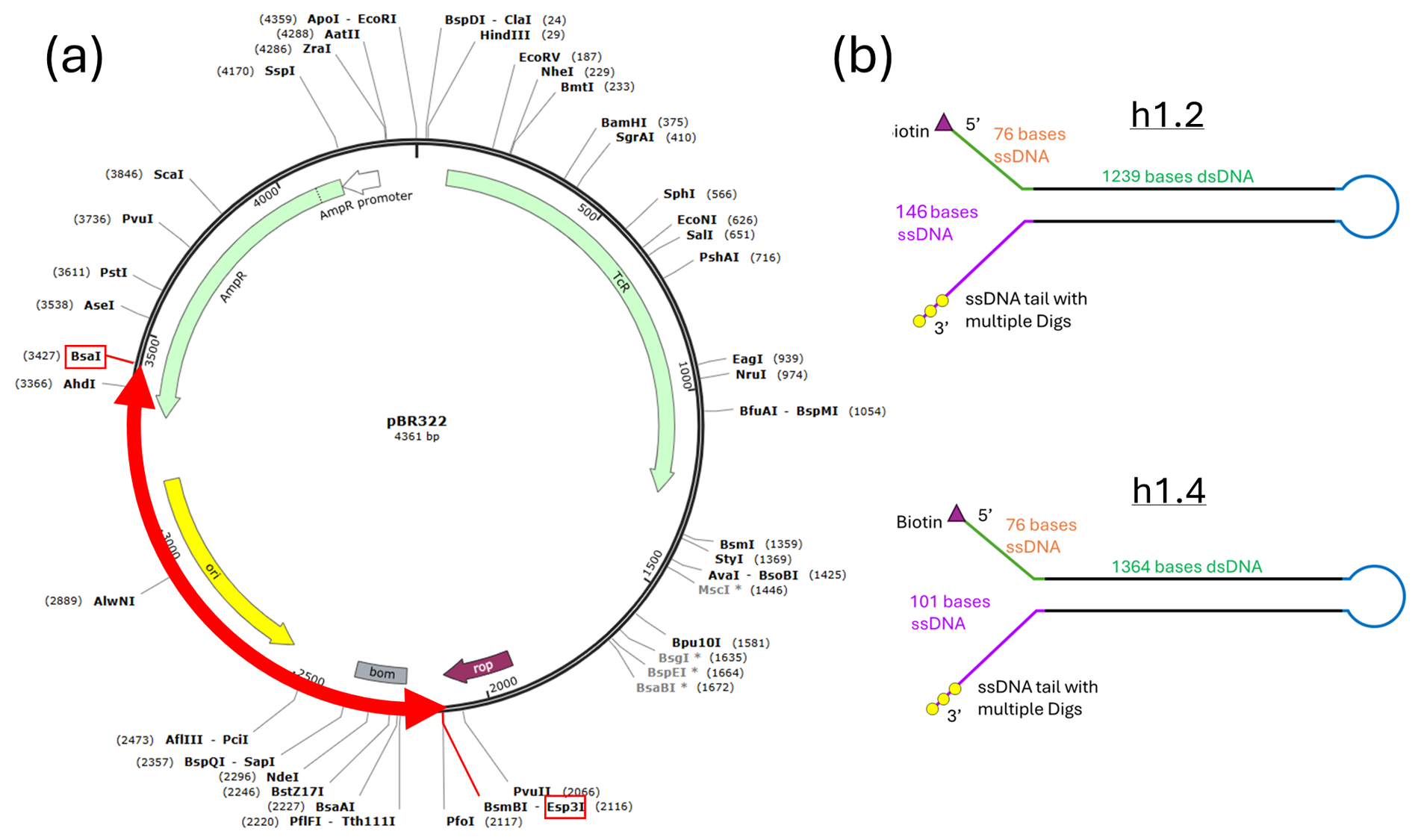

Supplement: Rodríguez-Franco et al. supplementary material [file S2633289225100112sup001.zip › Supplementary/Substrate_preparation.png]

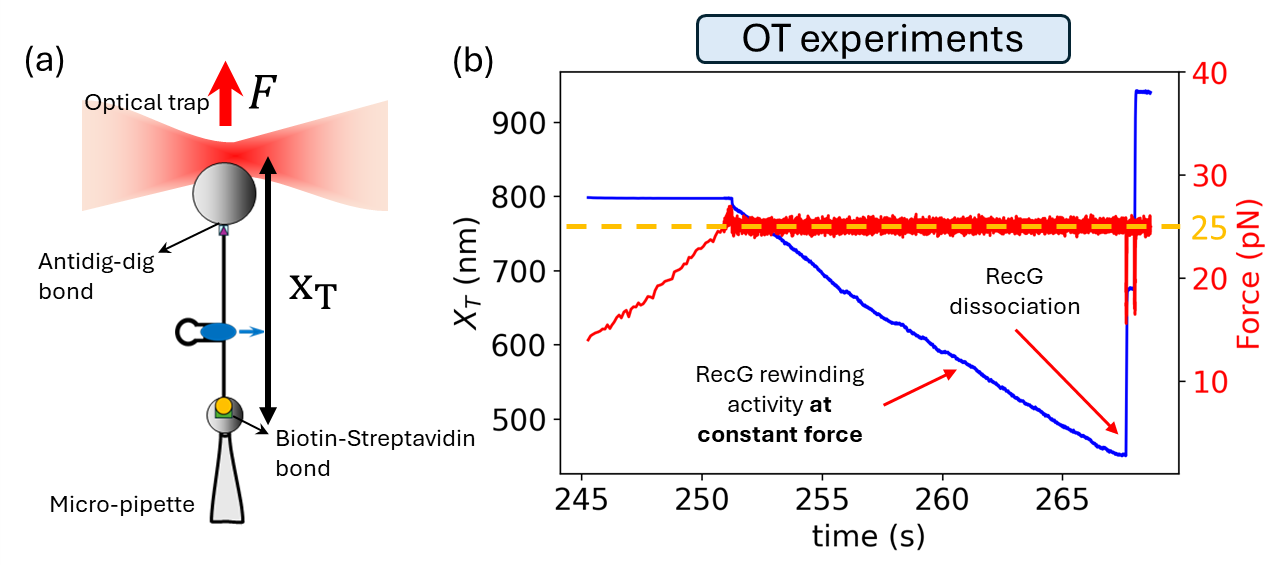

Supplement: Rodríguez-Franco et al. supplementary material [file S2633289225100112sup001.zip › Supplementary/optical_tweezers_experiments.png]

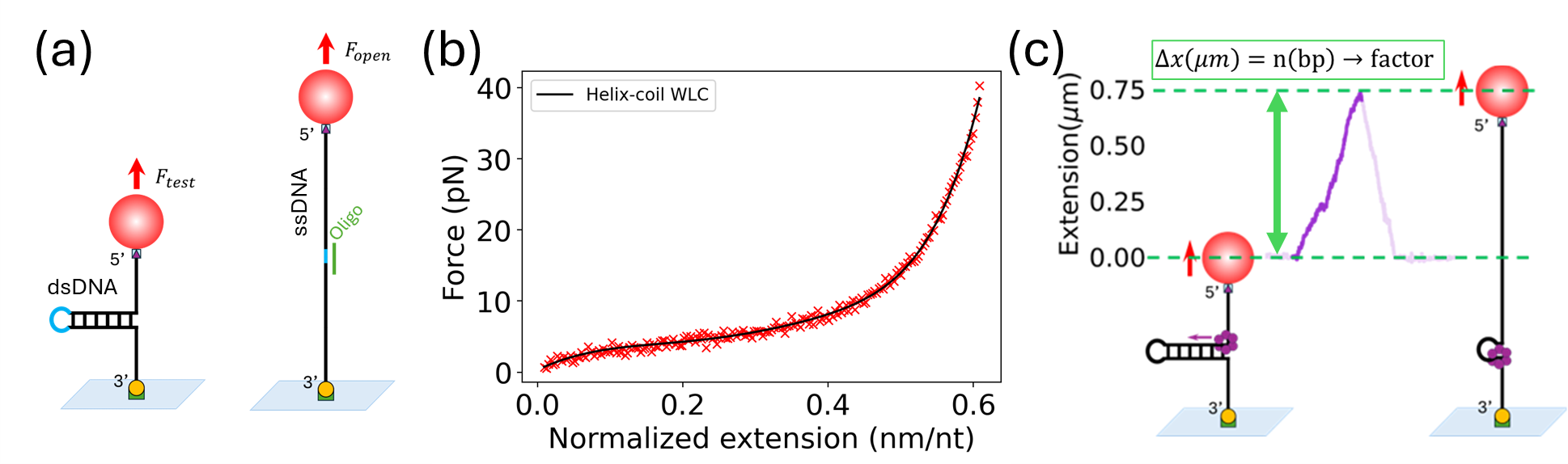

Supplement: Rodríguez-Franco et al. supplementary material [file S2633289225100112sup001.zip › Supplementary/factor.png]

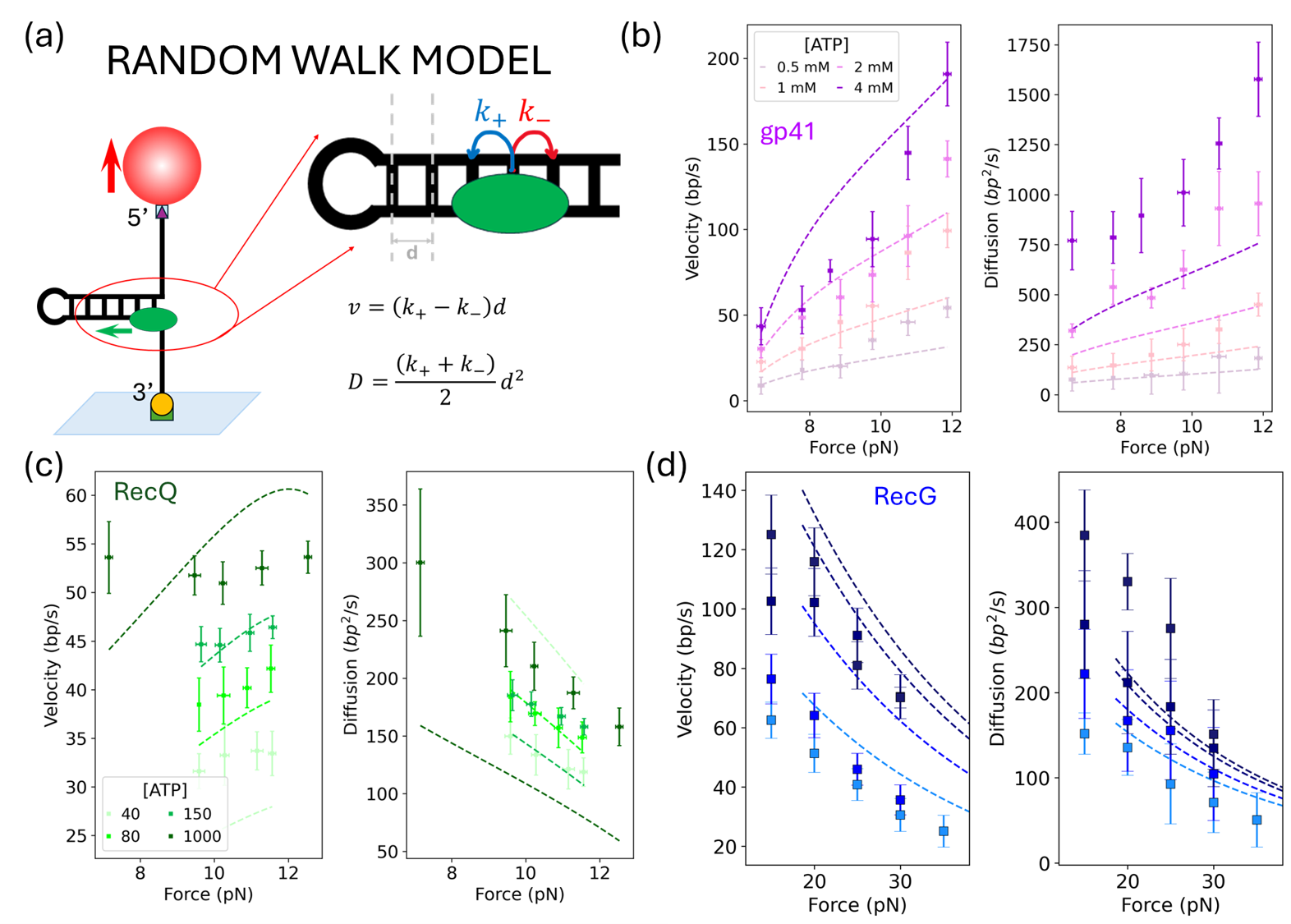

Supplement: Rodríguez-Franco et al. supplementary material [file S2633289225100112sup001.zip › Supplementary/rw_multifit.png]

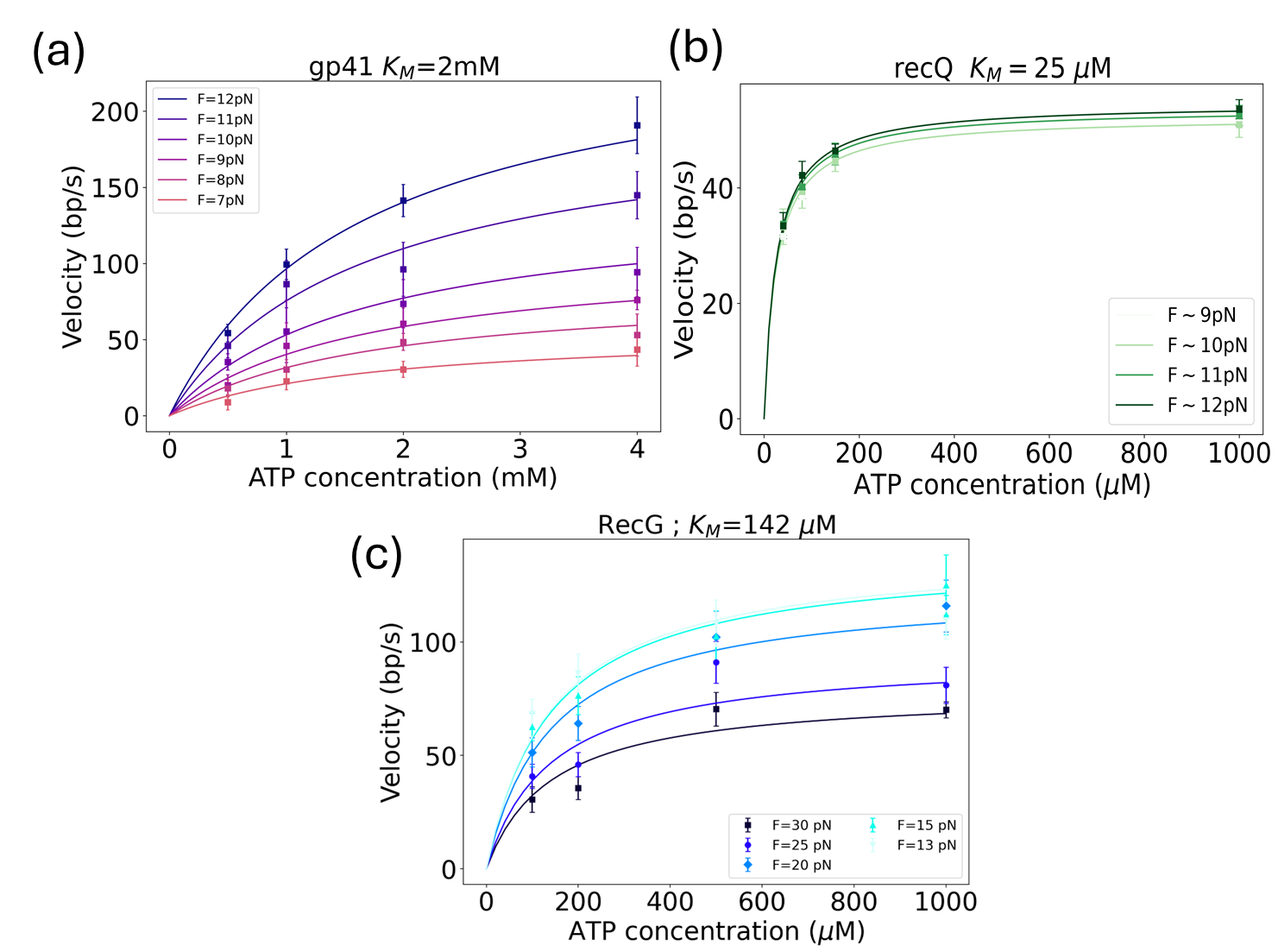

Supplement: Rodríguez-Franco et al. supplementary material [file S2633289225100112sup001.zip › Supplementary/Michaelis_Menten.png]

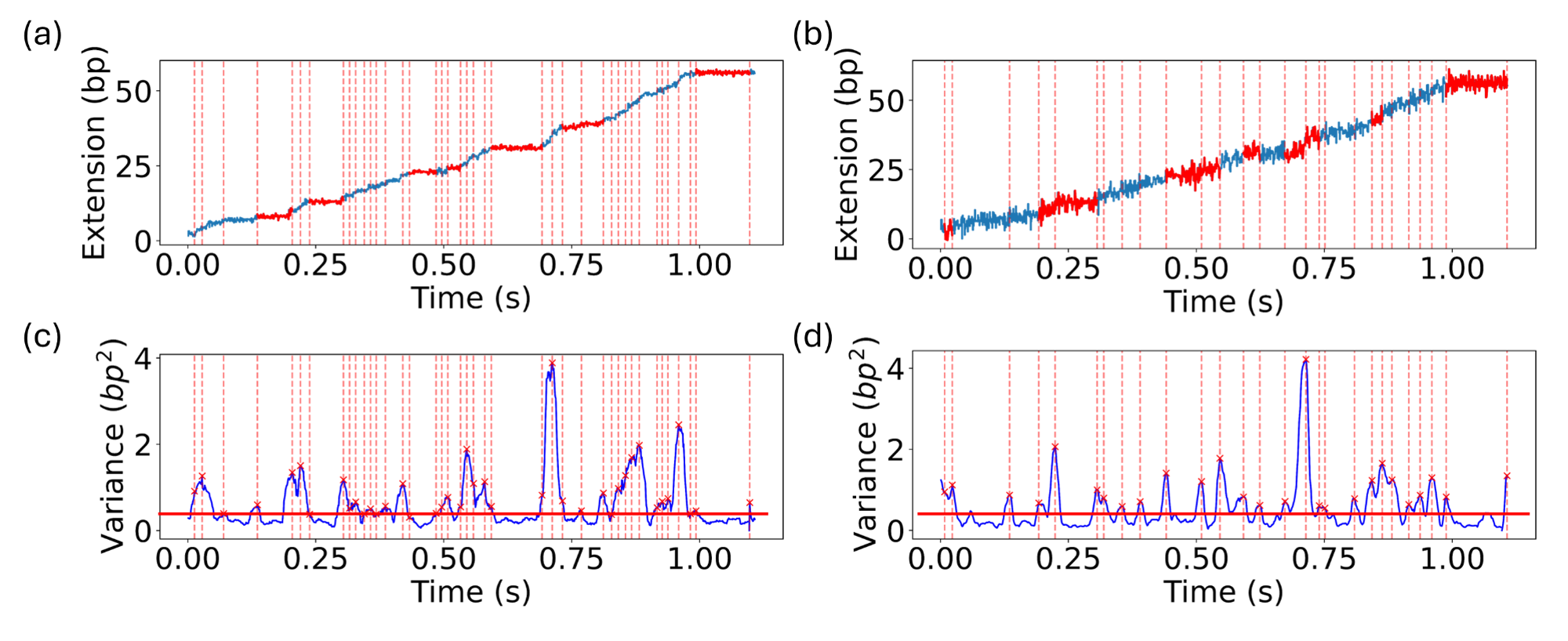

Supplement: Rodríguez-Franco et al. supplementary material [file S2633289225100112sup001.zip › Supplementary/pauses_1.png]

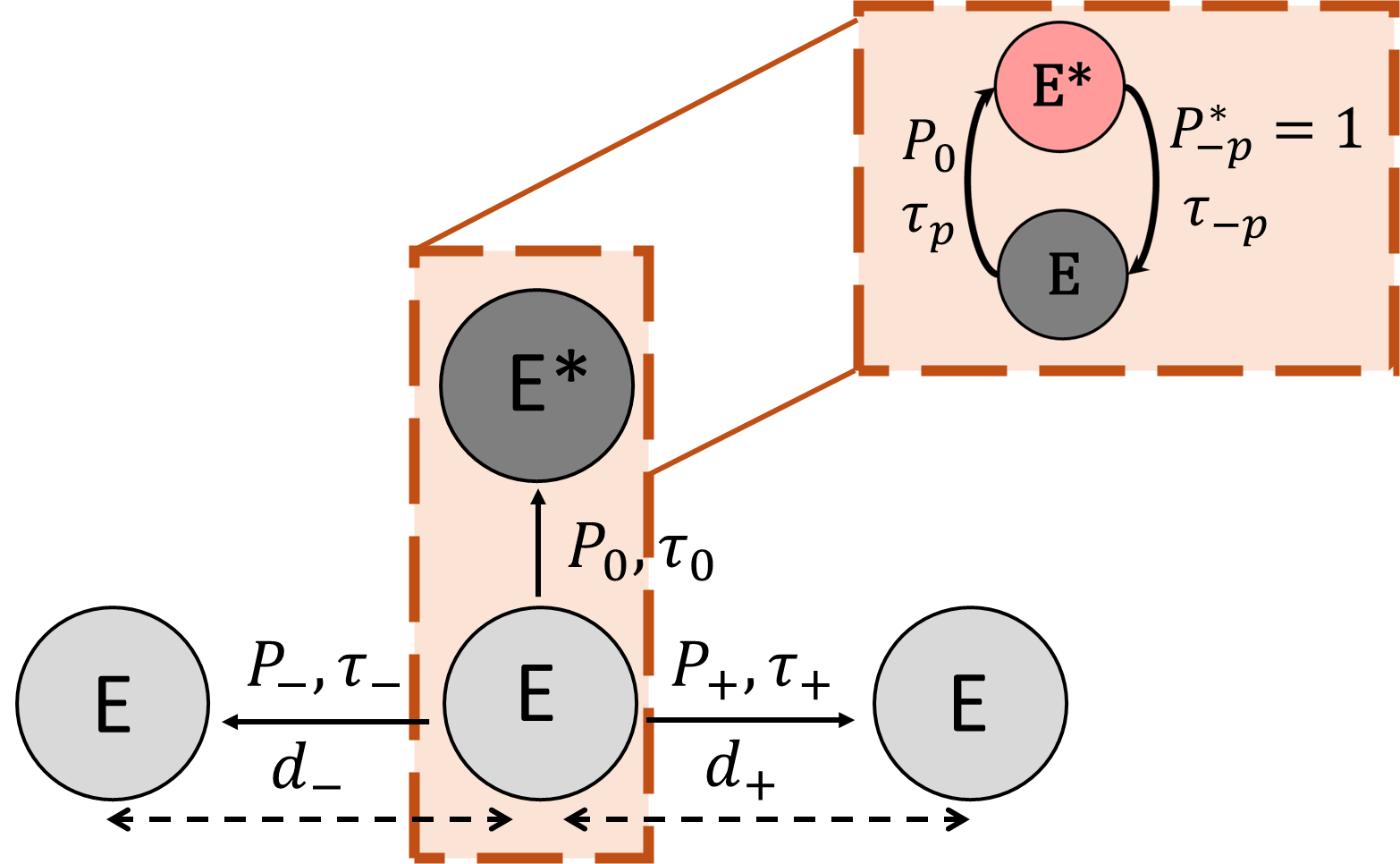

Supplement: Rodríguez-Franco et al. supplementary material [file S2633289225100112sup001.zip › Supplementary/model_PROBS.png]

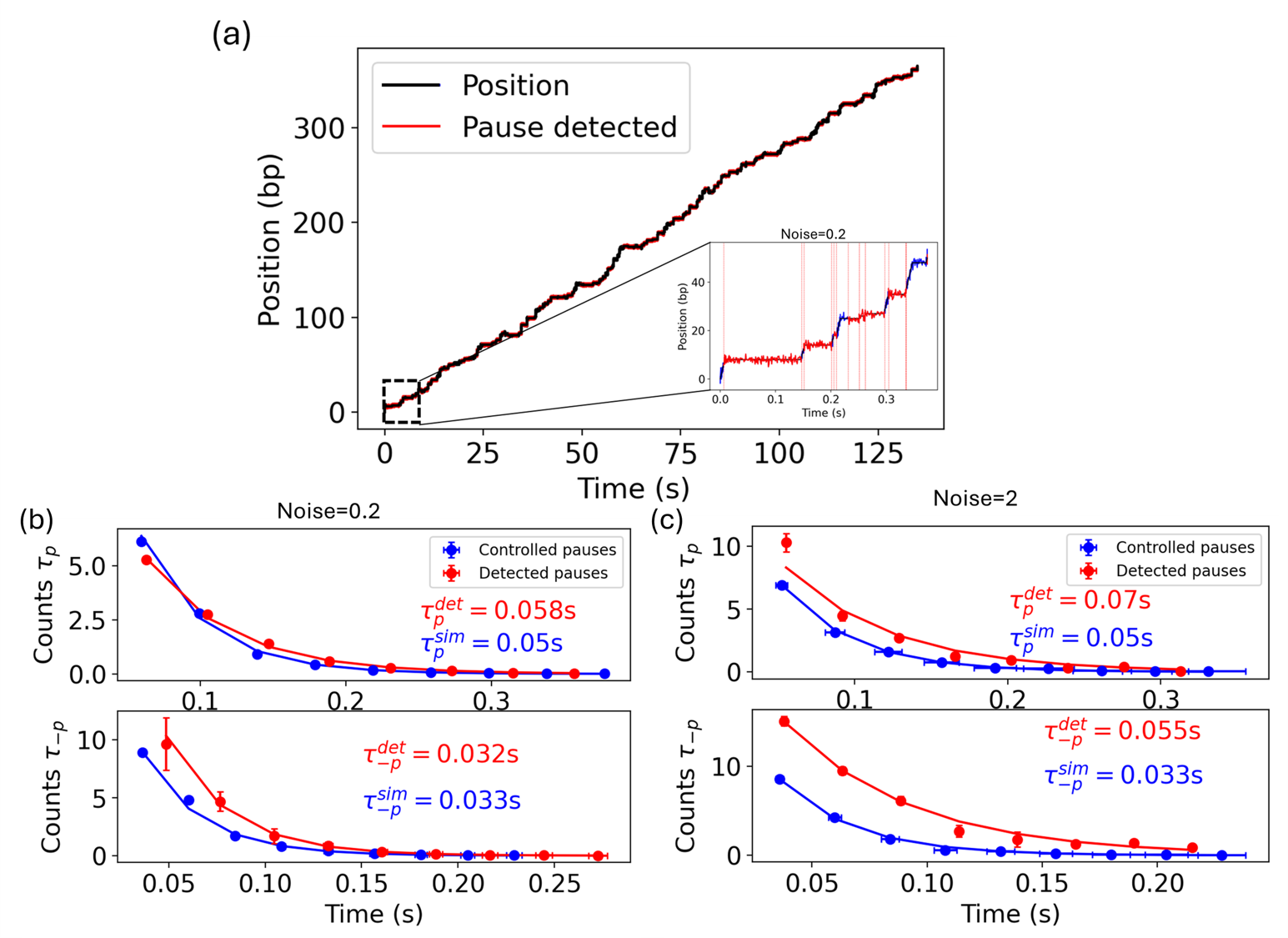

Supplement: Rodríguez-Franco et al. supplementary material [file S2633289225100112sup001.zip › Supplementary/pauses_detection.png]

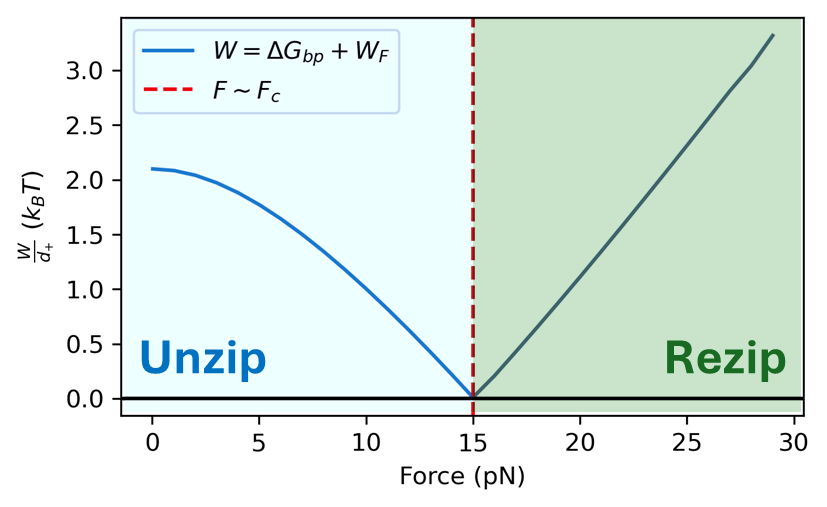

Supplement: Rodríguez-Franco et al. supplementary material [file S2633289225100112sup001.zip › Supplementary/Treball.png]

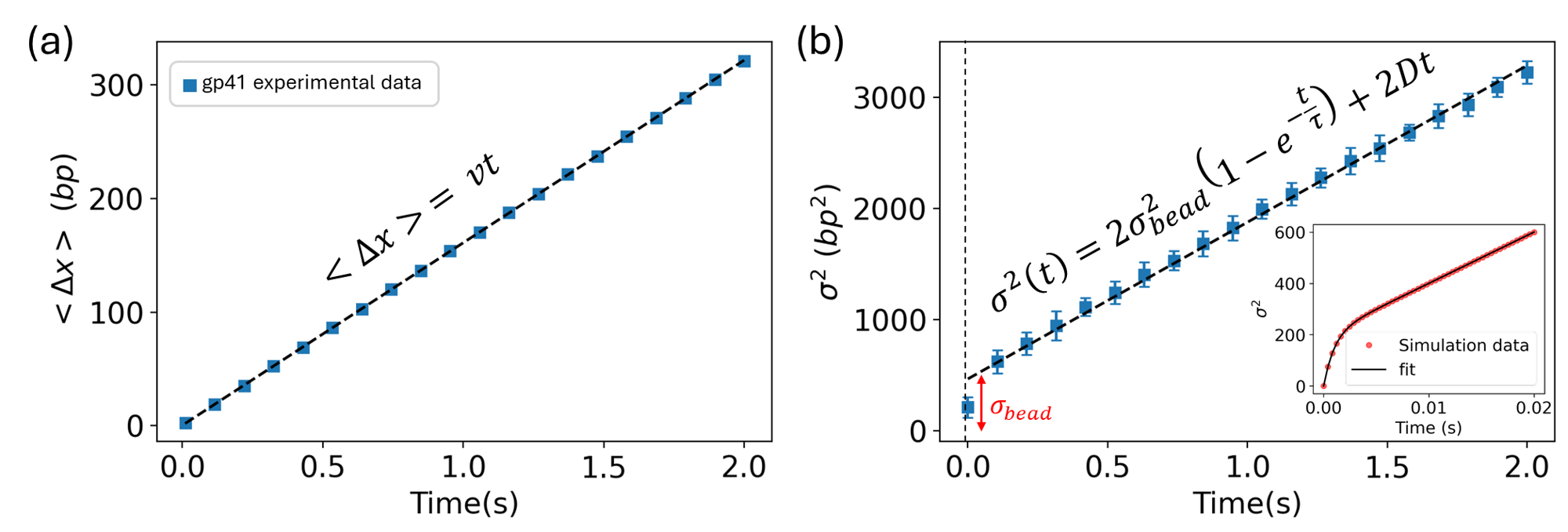

Supplement: Rodríguez-Franco et al. supplementary material [file S2633289225100112sup001.zip › Supplementary/variance.png]

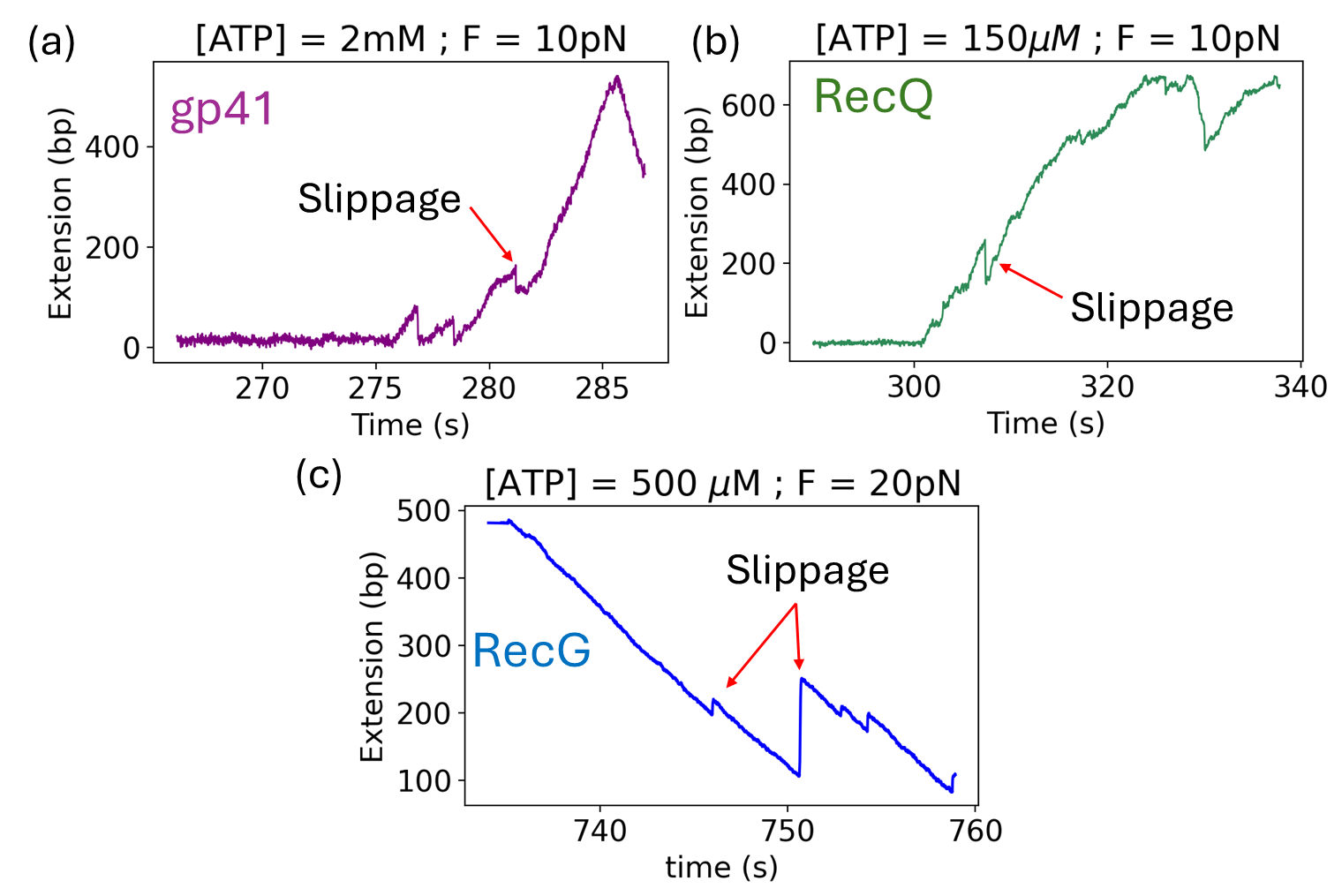

Supplement: Rodríguez-Franco et al. supplementary material [file S2633289225100112sup001.zip › Supplementary/Slippage.png]

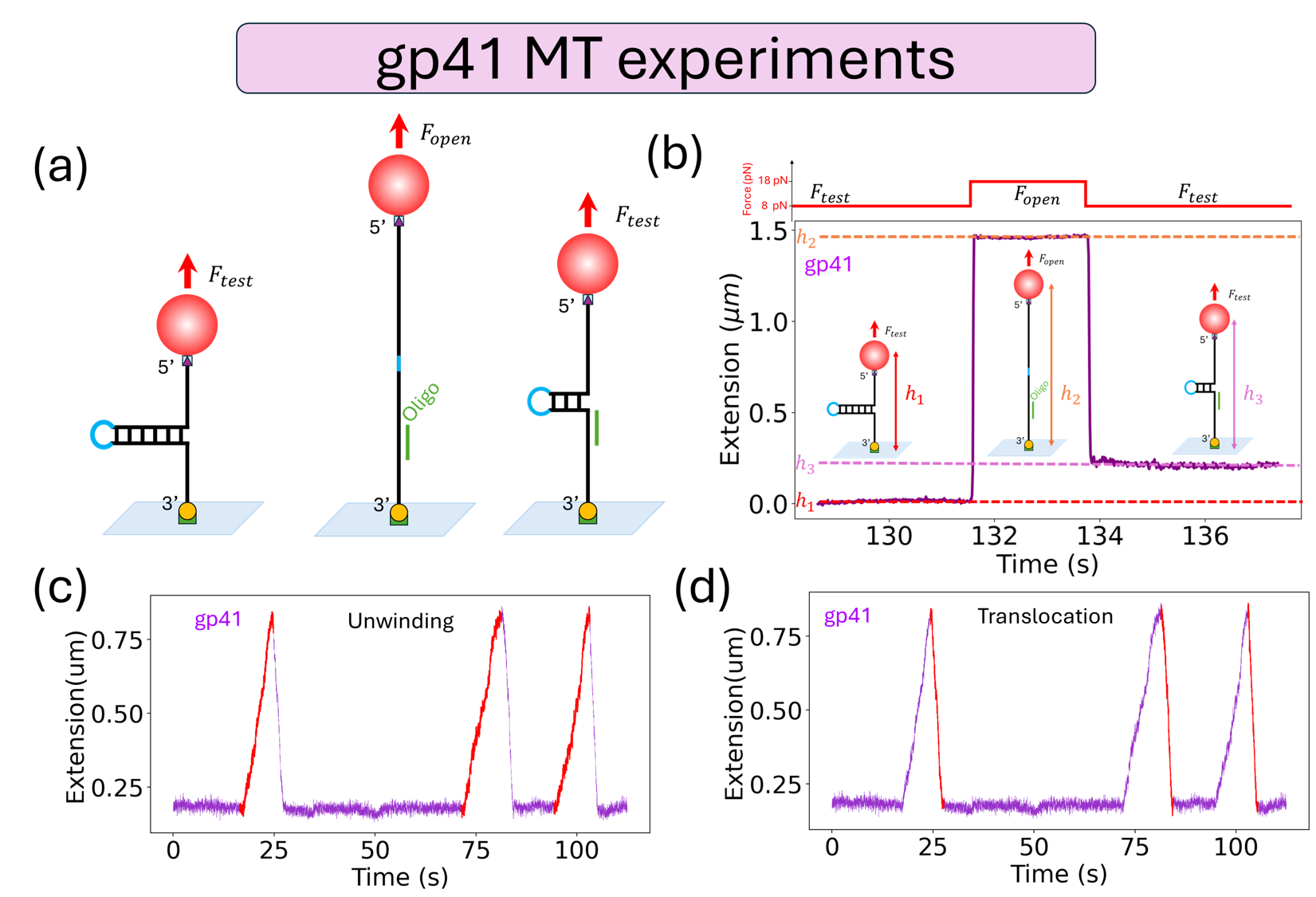

Supplement: Rodríguez-Franco et al. supplementary material [file S2633289225100112sup001.zip › Supplementary/gp41_oligos.png]

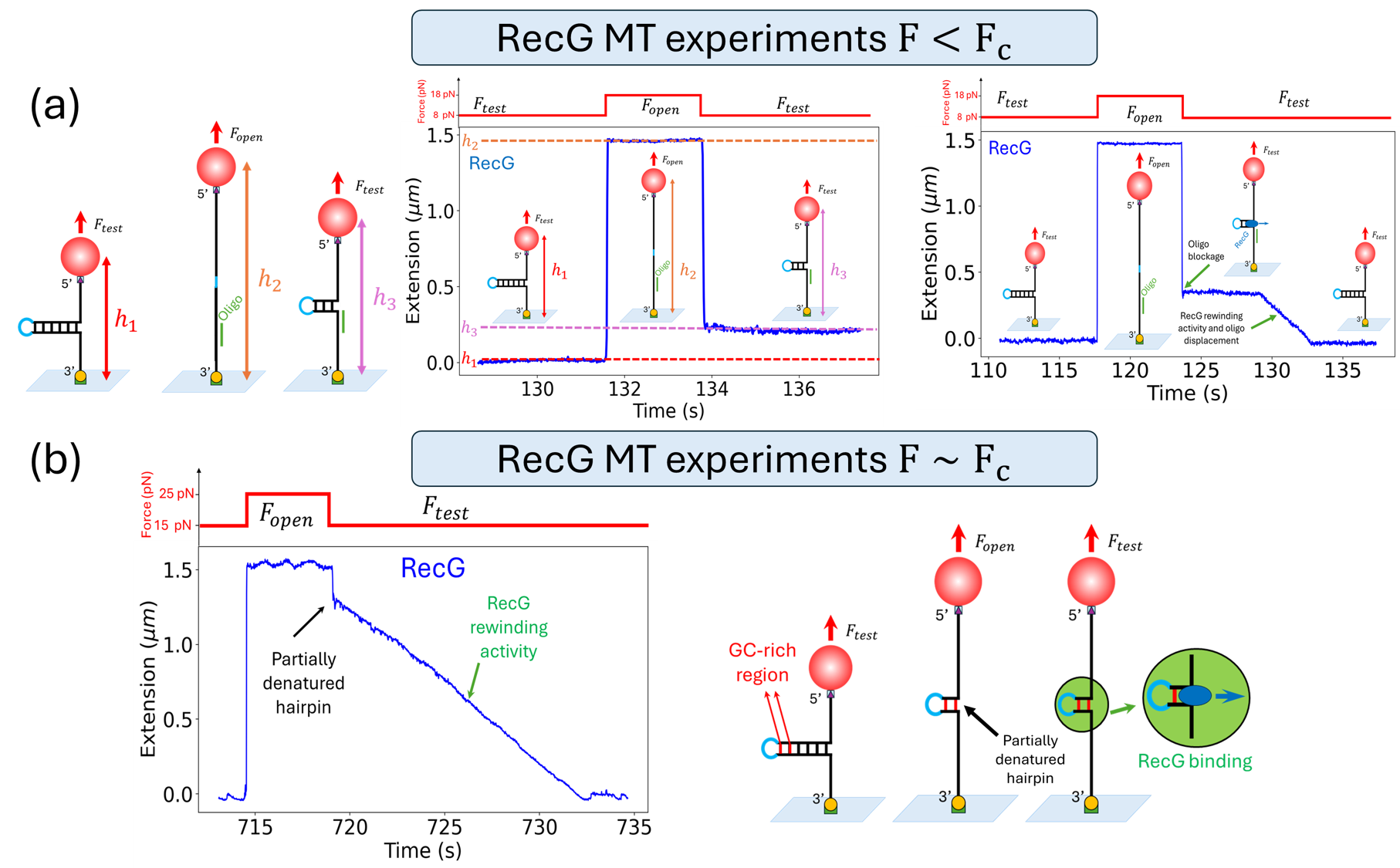

Supplement: Rodríguez-Franco et al. supplementary material [file S2633289225100112sup001.zip › Supplementary/RecG_oligos.png]

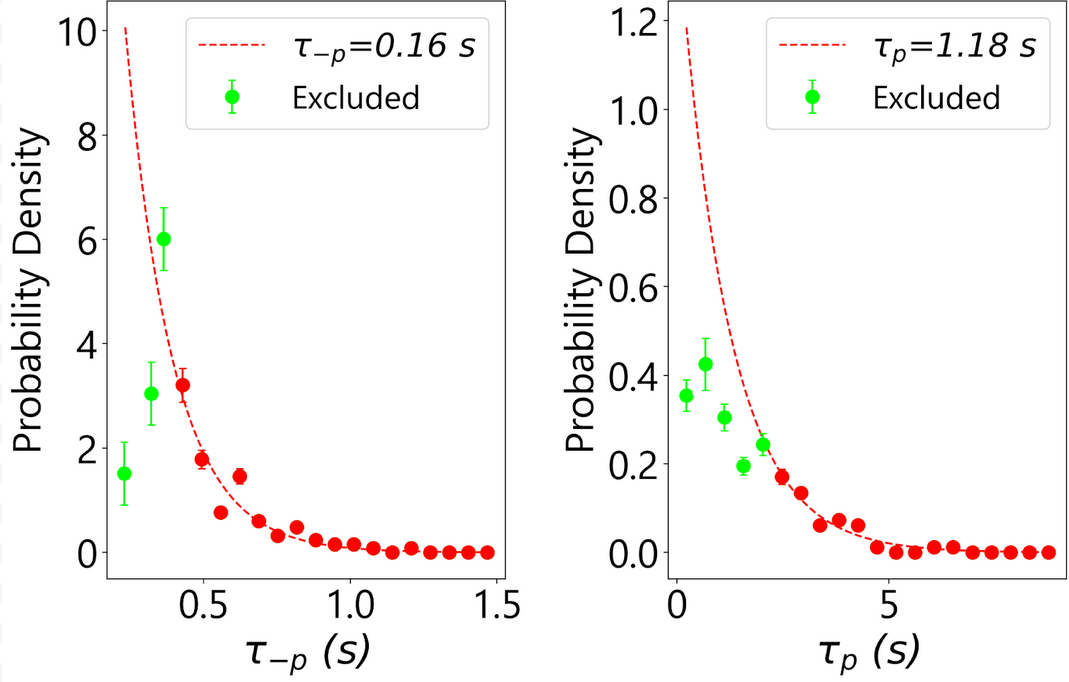

Supplement: Rodríguez-Franco et al. supplementary material [file S2633289225100112sup001.zip › Supplementary/Histogram_green_points.PNG]

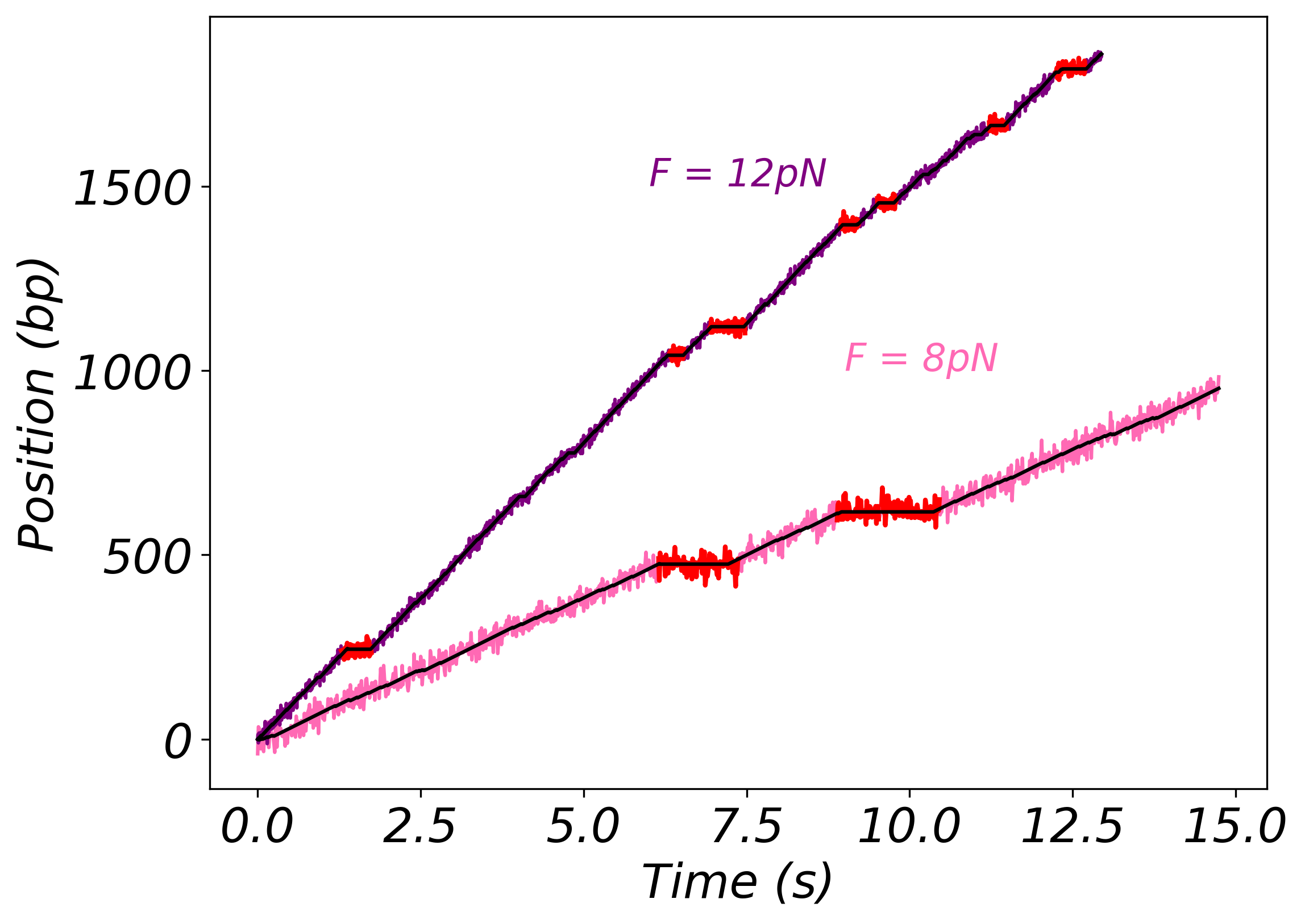

Supplement: Rodríguez-Franco et al. supplementary material [file S2633289225100112sup001.zip › Supplementary/simulation_gp41_noise.PNG]

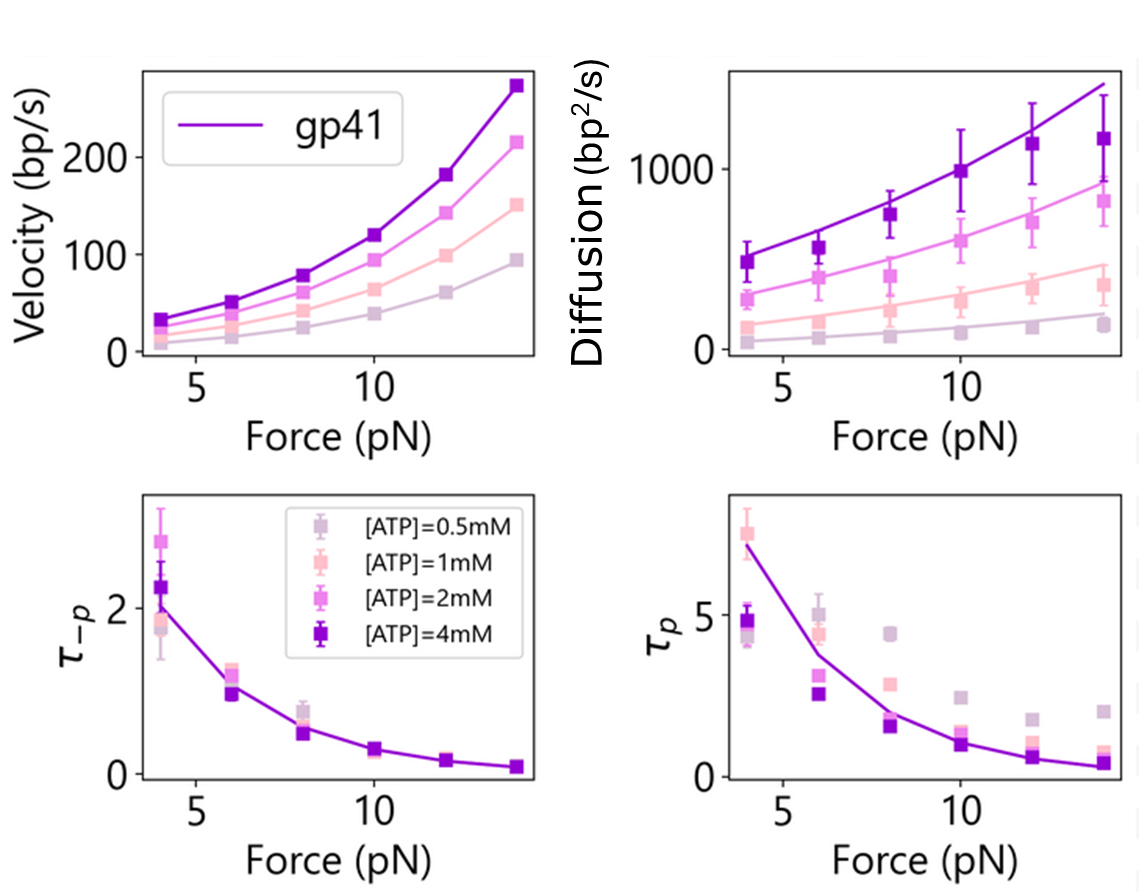

Supplement: Rodríguez-Franco et al. supplementary material [file S2633289225100112sup001.zip › Supplementary/gp41_simulation_results_MULTIFIT_plot.PNG]

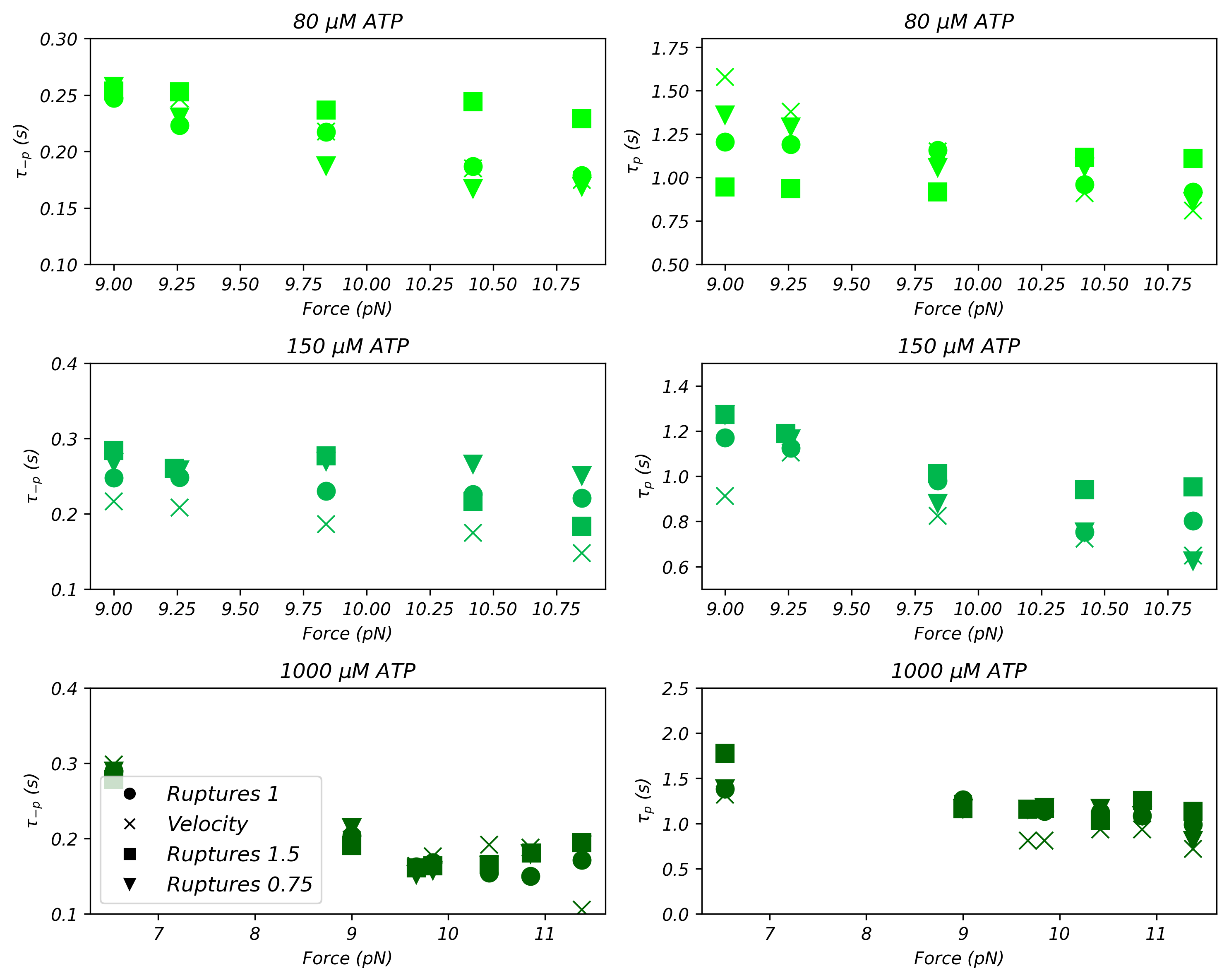

Supplement: Rodríguez-Franco et al. supplementary material [file S2633289225100112sup001.zip › Supplementary/RecQ_tau_diferent_algo.png]

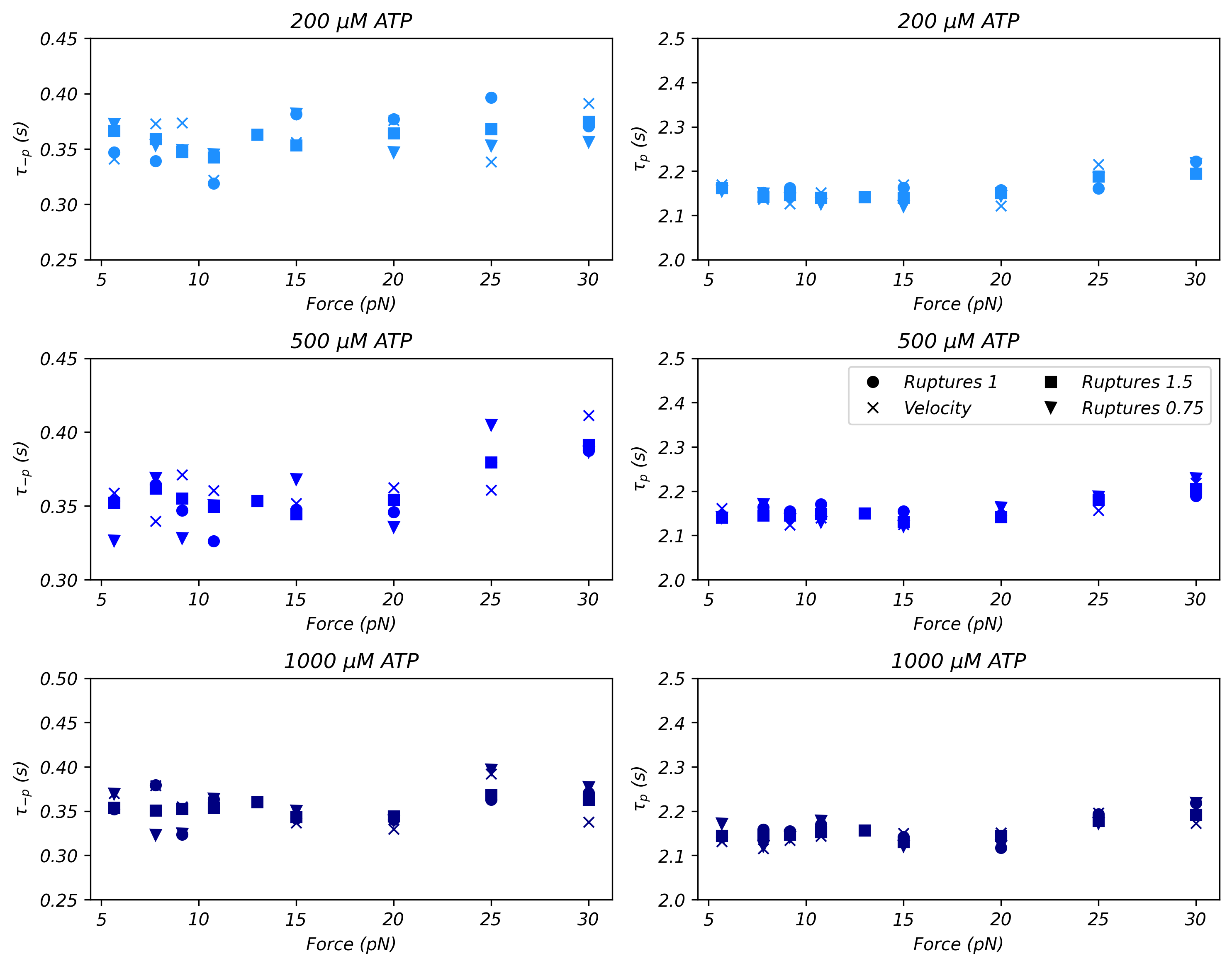

Supplement: Rodríguez-Franco et al. supplementary material [file S2633289225100112sup001.zip › Supplementary/RecG_tau_diferent_algo.png]

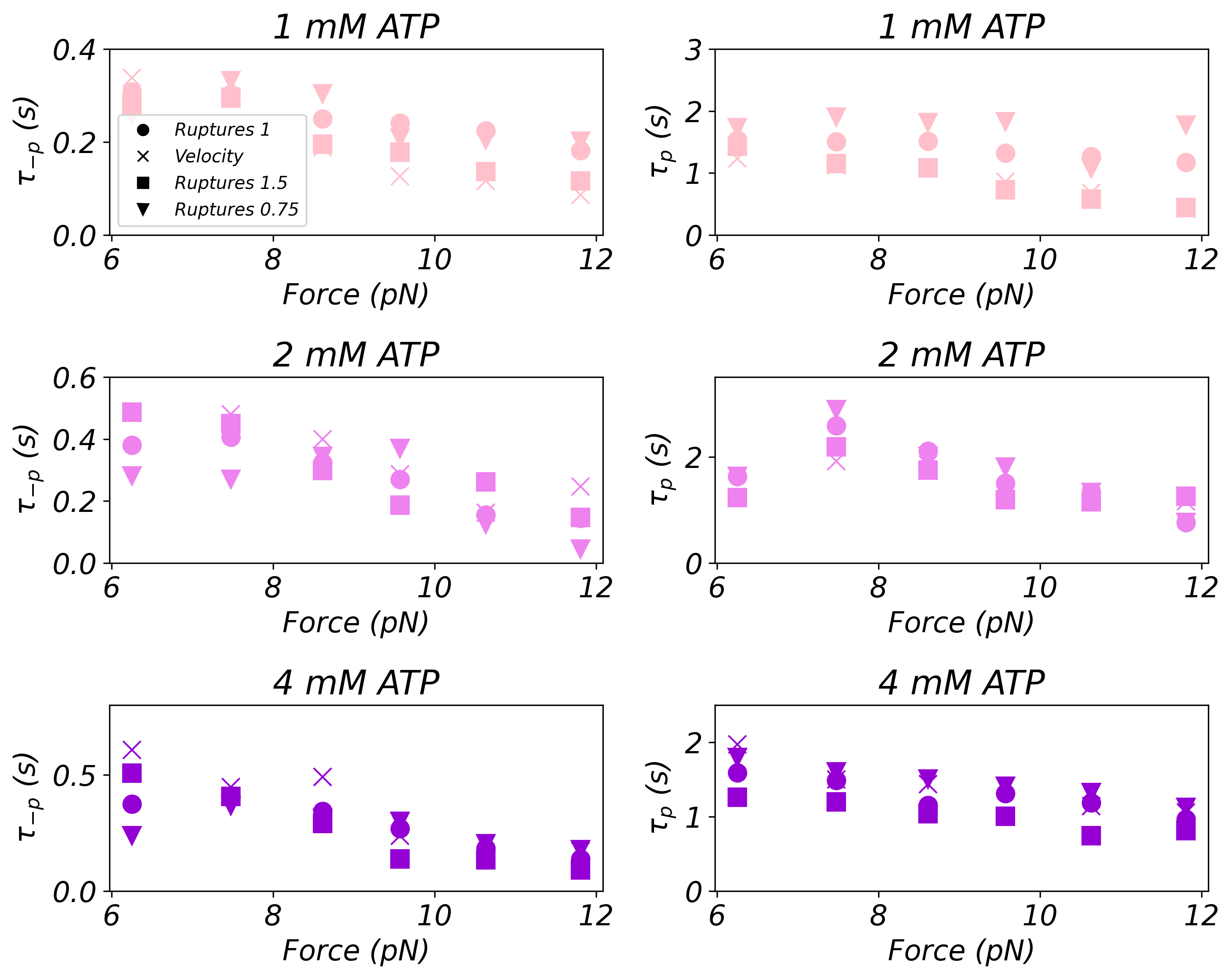

Supplement: Rodríguez-Franco et al. supplementary material [file S2633289225100112sup001.zip › Supplementary/gp41_tau_diferent_algo.png]

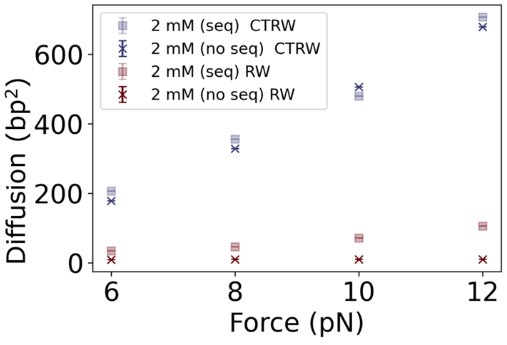

Supplement: Rodríguez-Franco et al. supplementary material [file S2633289225100112sup001.zip › Supplementary/sequencia_vs_no_sequencia.PNG]

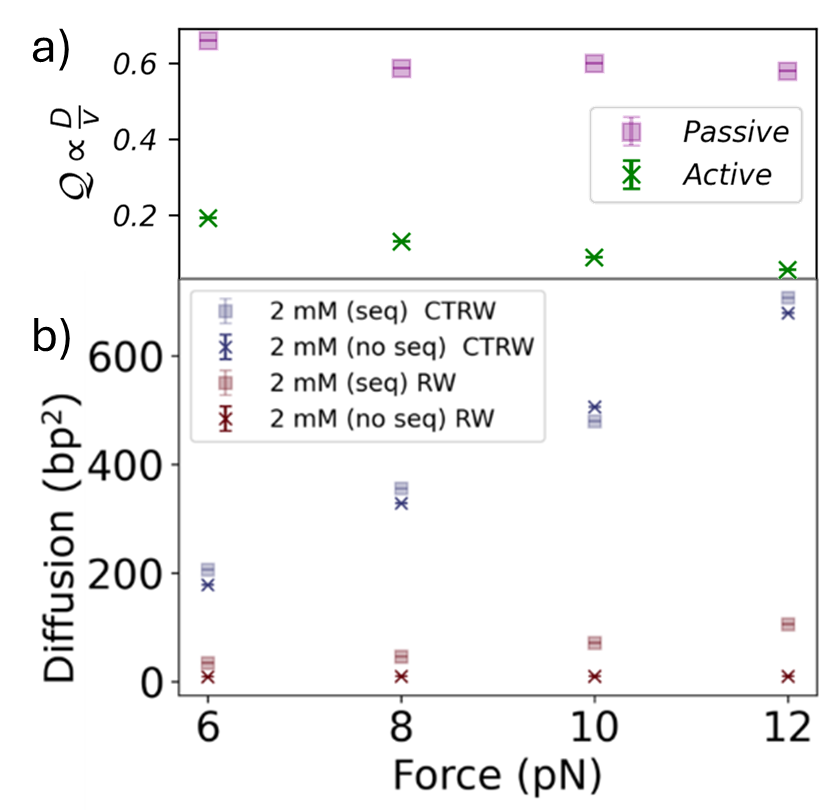

Supplement: Rodríguez-Franco et al. supplementary material [file S2633289225100112sup001.zip › Supplementary/factor Q passive and active.png]
